# Bacterial responses to plant antimicrobials: the case of alkannin and shikonin derivatives

**DOI:** 10.3389/fphar.2023.1244270

**Published:** 2023-08-07

**Authors:** Angélique Rat, Antigoni E. Koletti, Nebojša Rodić, Vassilios P. Papageorgiou, Anne Willems, Andreana N. Assimopoulou

**Affiliations:** ^1^ Laboratory of Microbiology, Department Biochemistry and Microbiology, Fac. Sciences, Ghent University, Ghent, Belgium; ^2^ Organic Chemistry Laboratory, School of Chemical Engineering, Aristotle University of Thessaloniki, Thessaloniki, Greece; ^3^ Natural Products Research Centre of Excellence (NatPro-AUTH), Center for Interdisciplinary Research and Innovation of AUTh, Thessaloniki, Greece

**Keywords:** shikonin, *Pseudomonas* sp., oligomers, metabolization, naphthoquinone, bacteria, metabolites, antimicrobial activity

## Abstract

Alkannin, shikonin and their derivatives (A/S) are secondary metabolites produced in the roots of certain plants of the Boraginaceae family such as *Lithospermum erythrorhizon* Siebold & Zucc. and *Alkanna tinctoria* (L.) Tausch. These naphthoquinones express anti-cancer, wound healing, and antimicrobial activities. To study the interactions between endophytic bacteria isolated from *A. tinctoria* and the antimicrobials A/S, endophytic bacteria known to be resistant to the compounds were screened for their effect on A/S in liquid medium. Thereafter, the strain *Pseudomonas* sp. R-72008, was selected and tested for its ability to modify A/S in nutrient medium and minimal medium with A/S as sole carbon source. Bacterial growth was recorded, and high performance liquid chromatography-diode array and ultra-high performance liquid chromatography-electrospray ionization-mass spectrometry analyses were performed to detect and quantify metabolites. In nutrient medium inoculated with R-72008, a decrease in the amount of A/S monomers initially present was observed and correlated with an increase of A/S oligomers. Moreover, a significant decrease of initial A/S monomers in minimal medium was correlated with bacterial growth, showing for the first time that a bacterial strain, *Pseudomonas* sp. R-72008, was able to use the naphthoquinones A/S as sole carbon source. This study opens new perspectives on the interactions between bacteria and plant antimicrobials.

## 1 Introduction

**FIGURE 1:**
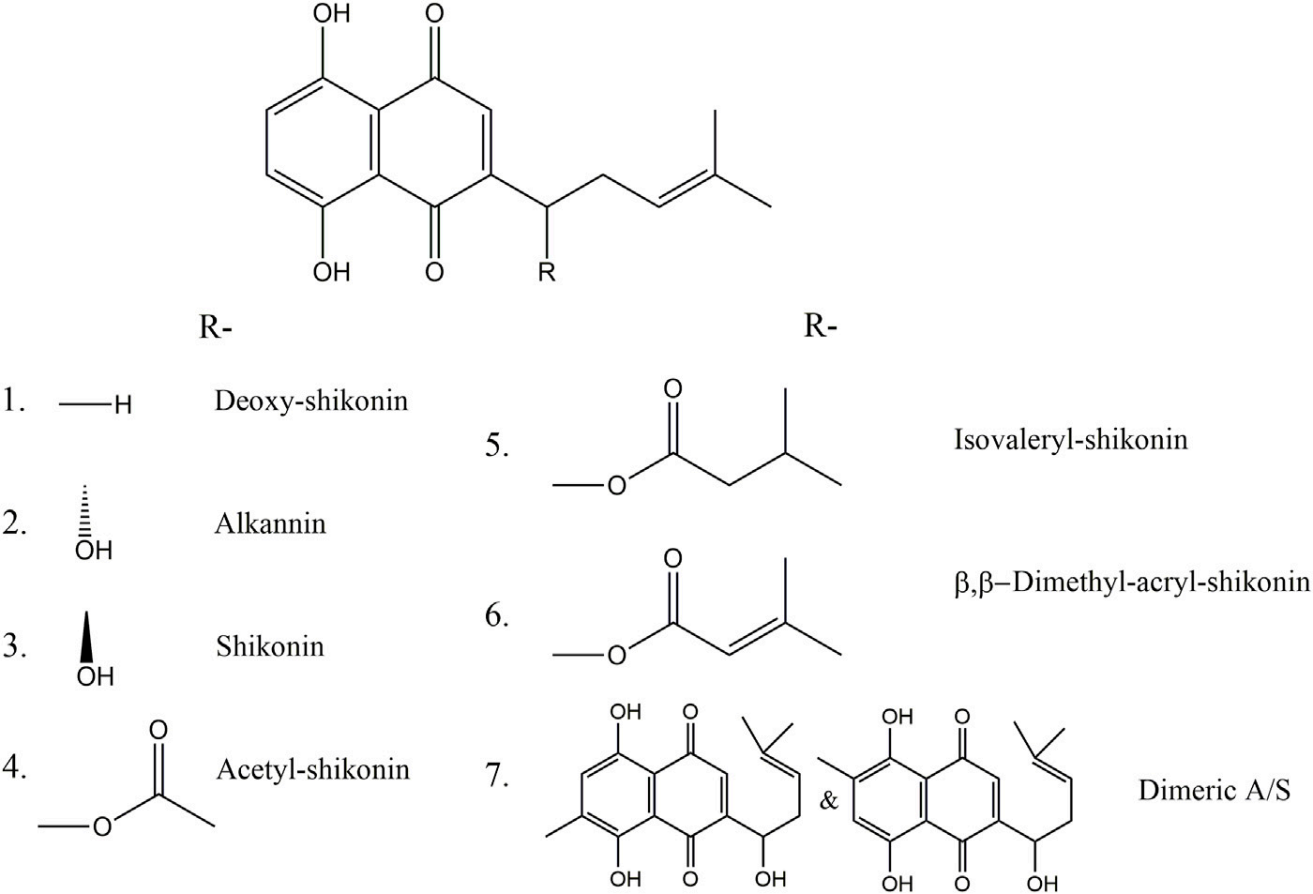
Chemical structures of (1) deoxy-shikonin, (2) alkannin, (3) shikonin, (4,5,6) shikonin ester derivatives and (7) alkannin/shikonin dimers.

Alkannin, its enantiomer shikonin, and their derivatives (A/S) are secondary metabolites belonging to the class of hydroxynaphthoquinones (Figure 1). They are natural lipophilic red pigments sensitive to pH that are red in acidic and blue in alkaline conditions (Papageorgiou et al., 1999). A/S are produced and secreted by root tissues in plants of the Boraginaceae family, such as *Alkanna tinctoria* (L.) Tausch (Alkanet) and *Lithospermum erythrorhizon* Siebold & Zucc., and their accumulation in the cork layer of mature roots leads to a red or purple coloration of the root (Singh et al., 2010; Tatsumi et al., 2016). The use of A/S goes far back in history with reports of their utilization as dyes and medicines during the Ming’s dynasty in China or in De Materia Medica by Dioscorides in Europe. However, these ethnopharmacological reports were only translated into scientific evidence after 1970, with the discovery of the science behind these ancient texts and subsequent revival of the use of Alkanet in therapeutics (Papageorgiou, 1978). This work led to the discovery and establishment of A/S derivatives as the active ingredients of *Alkanna tinctoria* (L.) Tausch roots with strong wound healing, regenerative, antimicrobial and anti-inflammatory activities, proved with clinical studies (Papageorgiou et al., 1999; Papageorgiou et al., 2008).

Nowadays, A/S receive an increasing interest for their pharmaceutical properties. Indeed, A/S are known for their wound healing, anticancer, regenerative, antioxidant and anti-inflammatory properties, as well as for their antimicrobial and antiviral activities (Gao et al., 2000; Sasaki et al., 2002; Chen et al., 2003; Assimopoulou et al., 2004; Papageorgiou et al., 2008; Yang et al., 2009; Li et al., 2012; Miao et al., 2012; Fu et al., 2013; Yadav et al., 2022). As many antimicrobials, A/S act on bacteria by disturbing the integrity of the bacterial membrane, by acting on the biosynthesis of essential bacterial proteins or by inhibiting their activities leading to cell death. For example, shikonin was shown to be efficient against virulent strains of *Staphylococcus pneumoniae*, *Candida albicans*, and the methicillin-resistant *Staphylococcus aureus* (Miao et al., 2012; Lee et al., 2015; Zhao et al., 2017). More recently, the potential of A/S against SARS-CoV-2 has been highlighted (Woo and Das, 2022).

The modes of action of A/S remain elusive but they have been suggested to be linked to their redox potential and ability to form of shikonin semiquinone radicals by reaction with dioxygen. The antioxidant activity of A/S has been associated with their ability to scavenge superoxide anion radicals, resulting in the formation of semiquinone radicals. The semiquinone radicals are then able to act directly as antimicrobials or to keep reacting with dioxygen to form superoxides or hydrogen peroxides. Moreover, A/S compounds can act as antimicrobials by chelating iron, causing starvation of iron-dependent microorganisms, and limiting the formation of biofilms (Sekine et al., 1998; Gao et al., 2000; Papageorgiou et al., 2008; Kumagai et al., 2012).

However, if A/S seem to be efficient against Gram-positive bacteria, several Gram-negative bacterial strains showed resistance to the compounds (Papageorgiou, 1980; Brigham et al., 1999; Rat et al., 2021). The resistance of bacteria to antimicrobials involves several mechanisms and resistance can be acquired by mutations and/or can be transferred among different bacterial species by horizontal gene transfer under antimicrobial pressure (Ventola, 2015). The resistance can involve the activation of efflux pumps and the modification of bacterial structures leading to lower affinity with antibiotic compounds. They can also act on antimicrobials by modifying their chemical structures causing the inactivation or even destruction of the compound. Indeed, bacteria are able to produce enzymes that will modify the functional groups of a molecule, leading to the inactivation of its antimicrobial property. For example, the production of β-lactamases results to the inactivation of β-lactam ring compounds such as penicillin, amoxicillin, and ampicillin (Shin et al., 2018; Khameneh et al., 2019). More specifically, Gram-negative bacteria possess an outer membrane which is mainly responsible for their resistance to many antimicrobials. As most antibiotics must pass the outer membrane to access their targets, the lack of this additional layer in Gram-positive bacteria makes them more sensitive to antibiotics (Breijyeh et al., 2020). This sensitivity is often related to the peptidoglycan cell wall of the Gram-positive bacteria, which is permeable to molecules with molecular weights in the range of 30,000–57,000 Da, allowing for the entry of many small antimicrobials, including A/S (Lambert, 2002).

As A/S have a great potential as antibiotics, understanding how the plant secondary metabolites A/S interact with Gram-negative bacteria is essential to use these compounds efficiently, the present work aimed at: 1) screening bacteria for A/S degrading activity, and 2) assessing how bacteria act on A/S compounds and what type of metabolites are formed during this interaction.

## 2 Materials and methods

### 2.1 Screening of A/S degrading bacteria

In order to gain insight into the bacterial mechanism of resistance to A/S, 53 strains were tested for their ability to act on a mixture of these compounds (red pigments). A mixture of A/S pigments was used that contained the following monomers: alkannin/shikonin, acetyl-alkannin/shikonin, deoxyshikonin, β,β-dimethylacryl-alkannin/shikonin, and isovaleryl-alkannin/shikonin; prepared as described in Assimopoulou et al. (2009). The LC-DAD and LC-MS analysis of the mixture of A/S pigments is depicted in Supplementary Figure S1. Among the bacteria tested, 29 strains had been isolated from *Alkanna tinctoria* (L.) Tausch and belonged to different Gram-negative genera (Rat et al., 2021) and 24 were *Pseudomonas* reference strains (LMG 2210, LMG 2152, LMG 2352, LMG 5060, LMG 5090, LMG 5093, LMG 5694, LMG 13184, LMG 21995, LMG 23076, LMG 26839, LMG 26898, LMG 27394, LMG 27930, LMG 28435, LMG 28439, LMG 28456, LMG 28495, LMG 28558, LMG 30013, LMG 30275, LMG 30830, LMG 30182, LMG 31089) obtained from the Belgian Co-ordinated collections of microorganisms, Bacteria Collection (BCCM/LMG). These LMG strains originated from various sources and were added to the dataset because, among the bacteria expressing resistance toward A/S in the study of Rat et al. (2021), several strains belonged to the genus *Pseudomonas*, and therefore we aimed to study whether the activity expressed by *Pseudomonas* strains toward A/S is specific of the ones isolated from A/S-producing plants. The assay was conducted in 96-wells flat-bottom plates containing, per well, 145 µL of medium R2B (Difco) supplemented with 250 mg/L of A/S mixture. Five microliters of a 24 h-bacterial culture (OD 0.4–0.6) resuspended in the medium R2B + A/S were then inoculated in triplicate. The assay was repeated twice. Uninoculated controls were also prepared for each medium. The plates were then incubated at 28°C, while shaking at 100 rpm, for 24 h. Bacterial effect on the compounds and bacterial growth in the medium was inspected visually to observe color changes and spectrophotometric analysis was performed at 300–800 nm and compared to uninoculated controls. Absorbance peaks at 520–580 nm correspond to A/S.

### 2.2 Degradation assay

Among the strains showing a difference compared with the uninoculated controls, one strain, *Pseudomonas* sp. R-72008, was chosen to perform an assay recording the effect on A/S by LC-DAD and UPLC-ESI-MS/MS analyses (see below). Two media were tested for A/S degradation: R2B supplemented with 250 mg/L A/S, and minimal medium (mineral salt medium, MSM, 1.0 g/L (NH_4_)_2_SO_4_, 1.5 g/L K_2_HPO_4_, 0.5 g/L KH_2_PO_4_, 0.2 g/L MgSO_4_, 7·H_2_O, 1.0 g/L NaCl, pH 7) (Yang et al., 2017) supplemented with 250 mg/L A/S as sole carbon source.

Firstly, an overnight bacterial culture was prepared. For this, a bacterial suspension of the selected strain was made by inoculating two loops of biomass in 100 mL R2B medium. Subsequently, after homogenization, two tubes were filled each with 35 mL of this bacterial suspension and incubated overnight at 28°C, 100 rpm.

Following the overnight incubation, the bacterial growth was measured by spectrophotometry to confirm the OD was about 0.5. The tubes containing the bacterial cultures were then centrifuged, the supernatants were discarded, and the pellets were resuspended in 35 mL R2B + 250 mg/L A/S or 35 mL MSM + 250 mg/L A/S. One milliliter of these suspensions was used to assess the colony forming units in these initial bacterial cultures. Five milliliters of each bacterial suspension were then inoculated into Erlenmeyers containing 145 mL of the corresponding medium. For each medium, the assay was performed in triplicate. Uninoculated controls were also prepared in triplicate for each medium by adding 5 mL of the corresponding medium without bacteria. In addition, to follow the bacterial growth without the presence of A/S, Erlenmeyers with R2B, MSM and MSM + 250 mg/L glucose were inoculated with the bacteria and then incubated at 28°C, 100 rpm for 24 h.

At the time of the bacterial inoculation (t = 0) and after incubation (t = 24 h), the pH of each culture was checked. Moreover, 1 mL was sampled for bacterial quantification, and for purity check of the uninoculated controls. Three ranges of dilution in PBS per sample were prepared and dilutions 10^−2^ to 10^−8^ were plated. Three times 5 mL (technical replicates) of each Erlenmeyer were also removed and transferred to 15 mL tubes for chemical analysis. The samples for chemical analysis were centrifuged at 9,500 g and the supernatants were transferred to new tubes and stored at -25°C followed by lyophilization. The resulting pellets were frozen in liquid nitrogen. Then, 1 mL of methanol was added to the frozen pellets and the samples were submitted to sonication for 15 min, 47 kHz. The resulting solutions were then centrifuged at 14,000 rpm, 4°C for 10 min and the supernatant was transferred to 2 mL Eppendorf tubes. The samples were left open under fume hood for solvent evaporation.

### 2.3 Chemical analyses

The effect of bacteria on the A/S mixture was assessed with chemical analysis of both lyophilized supernatants and pellets by HPLC-DAD analysis. Prior to analysis, pellets were dissolved in 1 mL methanol and then filtered over a 0.22 μm syringe filter (Labfil, China). Lyophilized supernatants were suspended in 4.7 mL methanol, followed by sonication for 2 min in a sonication bath. Then samples were filtered over a 0.22 μm syringe filter.

To quantify the effect of bacteria on the A/S mixture, both lyophilized supernatants and pellet samples were submitted to chromatographic analysis, using an ultra-high performance liquid chromatography (UHPLC) system (ECS05, ECOM spol. Sr.o., Czech Republic) hyphenated with a diode array detector (ECDA2800 UV-Vis PDA Detector). The system comprised a quaternary gradient pump (ECP 2010H), a gradient box with degasser (ECB 2004), a column oven (ECO 2080) coupled with a diode array detector. The chromatographic separation took place in a Fortis SpeedCore reversed phase column (C18, 2.6 μm, 100 × 4.6 mm). A binary elution system was used with A: water and B: acetonitrile with 0.1% formic acid with total flow 1 mL/min. Initially (t = 0 min) B was 70%, at 8 min B was 100% and remained constant for 5 min. Then, the system was equilibrated for 3 min with the initial conditions. The injection volume was 10 μL and the detector was set at several wavelengths (520, 254, 275, 407, 205, 225, 490 and 600 nm) including 520 nm that is the characteristic wavelength of A/S. Data from HPLC analysis were processed using Clarity Chromatography Software v8.2 (DataApex Ltd.).

In order to quantify derivatives of alkannin and shikonin in all samples, a mixture of standards including shikonin (Ichimaru, Japan), acetyl-shikonin (ABCR GmbH, Germany), deoxy-shikonin (TCI, Belgium), β,β-dimethylacryl-shikonin (ABCR GmbH, Germany) and isovaleryl-shikonin (TCI, Belgium) (previously purified, and fully characterized by A.N. Assimopoulou and V.P. Papageorgiou as described in Tappeiner et al., 2014), was used. Solutions of the standard mixture in a concentration range 1–80 ppm were prepared and analyzed. The calibration curves for each compound, the limit of detection (LOD) and the limit of quantification (LOQ) are presented in the Supplementary Table S5. For the validation of the method, a standard mixture of each A/S derivatives at 40 ppm was used. Calibration curves were constructed for three consecutive days. The precision of the method was calculated as follows: for shikonin 0.69%, for acetylshikonin 1.67%, for deoxyshikonin 0.48%, for β,β-dimethylacryl-shikonin 1.10% and for isovaleryl-shikonin 1.32%. The intra-day reproducibility of the method was calculated for shikonin 0.91, for acetylshikonin 1.29, for deoxyshikonin 0.39, for b,b-dimethylacryl-shikonin 1.67 and for isovaleryl-shikonin 1.73. Moreover, the inter-day day reproducibility of the method was calculated shikonin 1.15, for acetylshikonin 1.84, for deoxyshikonin 1.13, for β,β-dimethylacryl-shikonin 2.08 and for isovaleryl-shikonin 2.19.

Specific samples (MSM_R72008_B2T2_24h, R2B_Control_B2T2_0h, R2B_R72008_B1T1_24h, R2B_R72008_B2T2_24h, R2B_R72008_B1T1_24h, R2B_R72008_B3T1_24h, R2B_R72008_B3T3_0 h and R2B_R72008_B3T3_24 h) of the degradation study were also subjected to UPLC-ESI-MS/MS for further investigation of the degradation products. The ultra-performance liquid chromatography system (Thermo Scientific™ LTQ Orbitrap XL™) was equipped with a pump (AccelaPump 1,250) and a controlled temperature autosampler (Thermo Fisher Scientific Inc., Germany). The chromatographic separation took place on an Acquity UPLC HSS C18 SB 1.8 μm, 2.1 × 100 mm column (Waters), the column temperature was 50°C and the injection volume was 5 μL. A binary solvent system used for the elution of the compounds consisted of A: Methanol with 0.1% formic acid and B: Water with 0.1% formic acid, at the flow rate of 0.3 mL/min. The elution gradient was at t = 0 min A: 5%, at t = 1 min A: 50%, at t = 8 min A: 100%, which remained constant for 5 min followed by 3 min post run time, in initial conditions, to fully equilibrate the column. The mass spectra were acquired in positive ionization mode after preliminary studies with both ionization modes. The data were analyzed using Xcalibur Data System (Thermo Fisher Scientific Inc., Germany).

### 2.4 Statistical analyses

Statistics were performed on the data obtained from HPLC-DAD in order to compare the effect of strain *Pseudomonas* sp. R-72008 on the total monomeric A/S content of the mixture. To be able to sum the total A/S content of the pellets and supernatants, the results were expressed in milligrams. Three biological replicates and three technical replicates per treatment were used. The normal distribution of the residuals was verified with the Shapiro-Wilk normality test. Then, the appropriate comparison test was performed. One-way ANOVA followed by a Newman Keuls test was conducted for residuals with normal distribution. Residuals non-normally distributed were compared with a Kruskal–Wallis test followed by a Dunn test with Benjamini–Hochberg adjustment. The statistical analyses were performed with the software R x64 4.1.0, using the packages agricolae and PMCMRplus.

## 3 Results

### 3.1 Screening of A/S degrading bacteria

**FIGURE 2:**
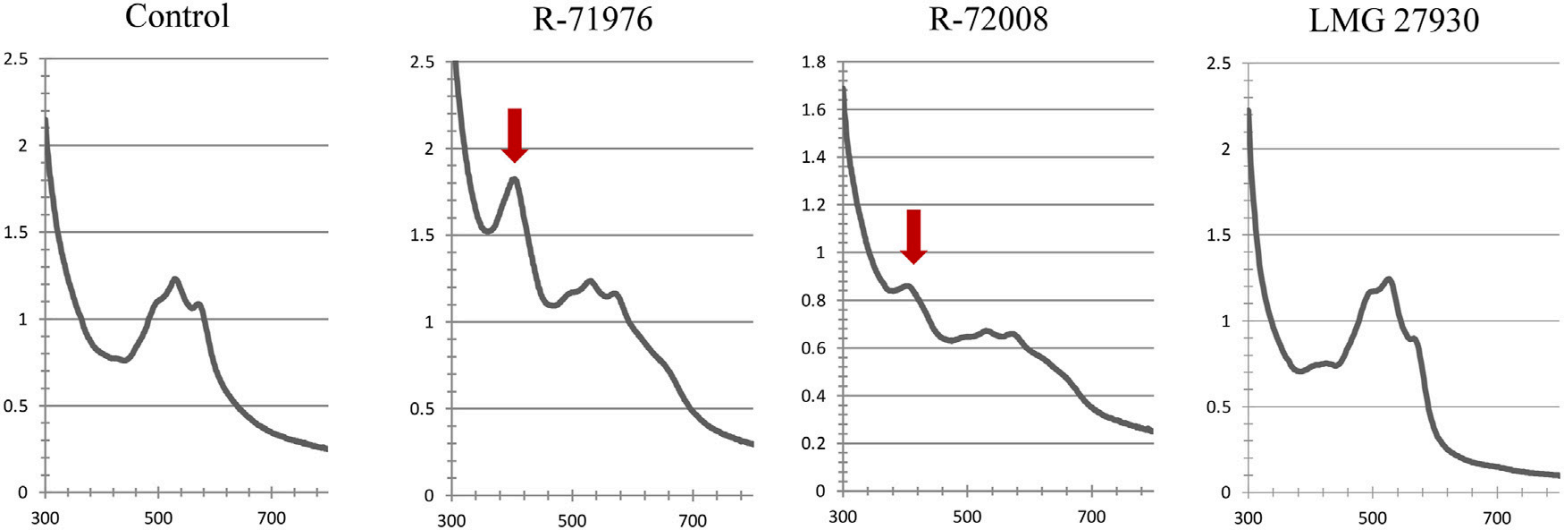
Absorbance spectra at 300–800 nm of R2B medium supplemented with 250 mg/L A/S. Characteristic A/S peaks at 520–580 nm. In presence of *Pseudomonas* sp. strains R-71976 or R-72008, a new peak at 410 nm appears, indicated by the red arrow. It is associated with a purple color of the medium. *Pseudomonas karstica* LMG 27930 showed the same spectral profile as the control and the same pinkish medium color.

A screening was first conducted to observe the effect of bacteria on the A/S absorbance. As A/S are red dyes, preliminary visual observations could be performed. Compared to the uninoculated controls, most of the wells inoculated with bacteria showed a change in color: the color changed mainly from dark pink to purple, but other colors could be observed such as light pink and brown (Supplementary Table S1). The spectrophotometric measurements associated with these observations indicated a reduction of the peak at 520–580 nm (characteristic for A/S) and the appearance of a new peak at 410 nm. The peak height at 410 nm varied between bacterial treatments. For example, comparing two *Pseudomonas* strains R-71976 and R-72008, the peak of R-72008 was much smaller (Figure 2). The strain LMG 27930 (*Pseudomonas karstica*) did not exhibit difference of color or spectrum with the uninoculated control. Interestingly, the height of the peak at 410 nm was not always well correlated with the absorbance value: some strains with a lower absorbance than the control, still showed a little peak at 410 nm (Supplementary Table S1).

### 3.2 Degradation assay

#### 3.2.1 Bacterial growth

Based on the preliminary screening, the strain *Pseudomonas* sp. R-72008 was selected for an assay on the degradation of A/S compounds. Indeed, during the screening, R-72008 was associated with a reduction of the peak at 520 nm, and the appearance of a small peak at 410 nm compared to other *Pseudomonas* sp. strains (see above). We hypothesized that this strain was responsible for degrading and consuming or converting A/S with the production of new compounds with absorbance at 410. To confirm this hypothesis, degradation assays were performed with R2B medium + 250 mg/L A/S and MSM medium with 250 mg/L A/S as sole carbon source.

The bacterial growth was recorded in both media supplemented with A/S and compared with MSM medium without supplement, MSM medium supplemented with 250 mg/L glucose and R2B medium without supplement. At t = 0, the bacterial concentrations in both MSM and R2B media were 3.30*10^6^ cfu/mL (SD: 4.98*10^5^) and 4.28*10^6^ cfu/mL (SD: 1.71*10^5^) respectively. After 24 h, the concentration of R-72008 in R2B was 6.33*10^8^ cfu/mL (SD: 5.86*10^7^) and 4.34*10^8^ cfu/mL in R2B + 250 mg/L A/S (SD: 3.81*10^7^). The strain R-72008 was growing as well with or without the presence of A/S in the R2B medium. In MSM medium without carbon source, the bacterial concentration decreased to 3.67*10^5^ cfu/mL (SD: 3.79*10^4^). In MSM medium supplemented with 250 mg/L A/S or 250 mg/L glucose, the bacterial concentration reached 2.45*10^8^ cfu/mL (SD: 1.81*10^7^) and 1.41*10^8^ cfu/mL (SD: 2.00*10^7^) respectively, showing the ability of the bacterium to use A/S as carbon source to support its growth in nutrient-poor medium.

#### 3.2.2 HPLC-DAD analysis

To understand the molecular processes involved when *Pseudomonas* sp. R-72008 is in the presence of A/S, a targeted metabolomics study was performed.

Peaks associated with monomeric A/S have retention times of less than 6 min: alkannin/shikonin (retention time, Rt = 1.73 min); acetyl-alkannin/shikonin (Rt = 2.79 min); deoxy-shikonin (Rt = 4.08 min); β,β-dimethylacryl-alkannin/shikonin (Rt = 4.96 min); and isovaleryl-alkannin/shikonin (Rt = 5.24 min). Peaks with retention time from 6 min to 12 min are associated with oligomeric A/S: as depicted by the LC-MS analyses conducted (same elution order with HPLC-DAD for C18 columns), peaks eluted at 6–8 min are associated with dimers; at 8–10 min to trimers, while after 10 min to other oligomers. Interestingly, the HPLC-DAD analysis revealed that the 407 nm wavelength, close to our peak of 410 nm (Figure 2) in the screening assay, was correlated with A/S oligomers absorbance spectra (Supplementary Figure S2E).

**FIGURE 3:**
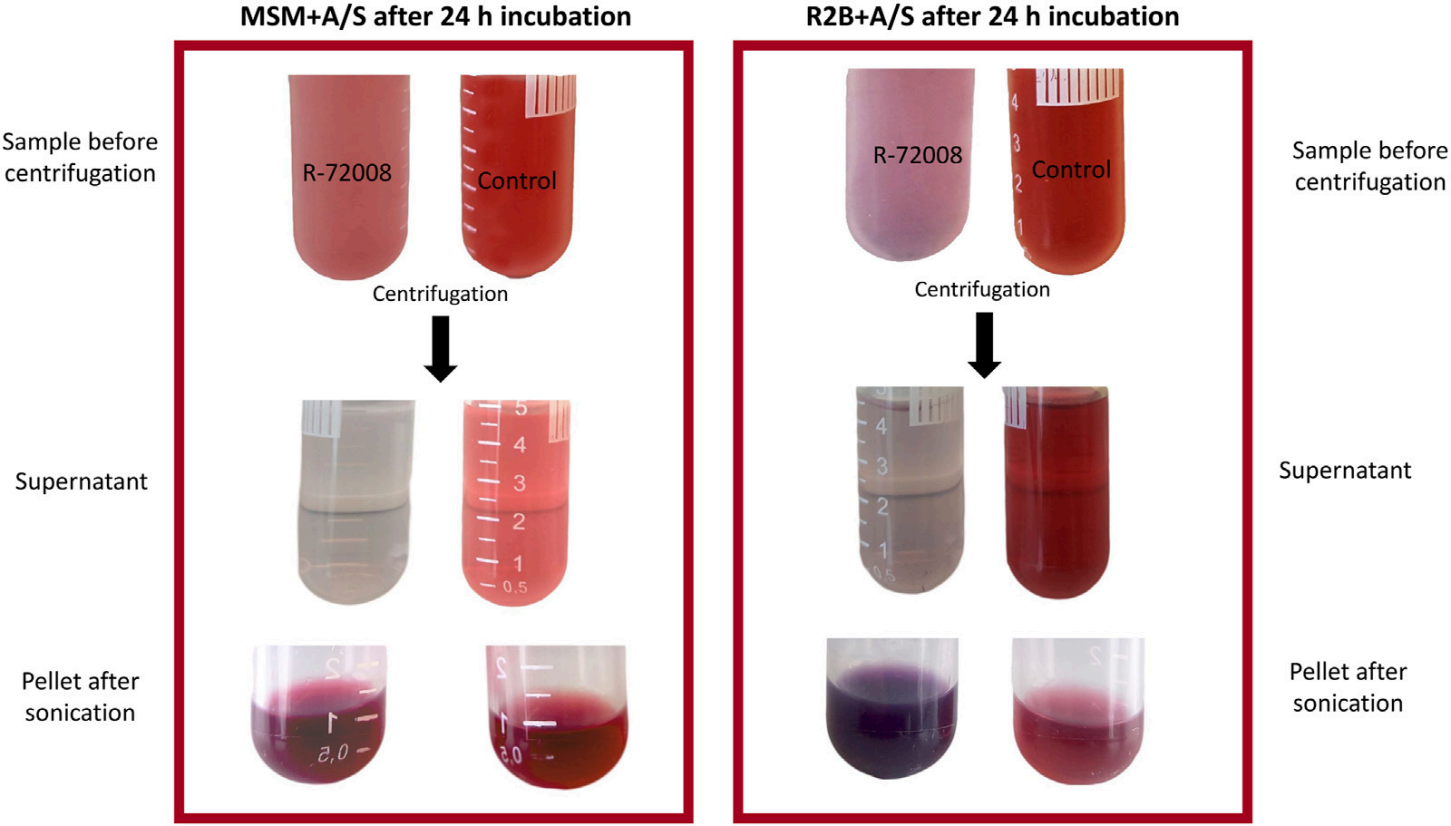
Effect of strain *Pseudomonas* sp. R-72008 grown in nutrient medium R2B and minimal medium MSM supplemented with 250 mg/L A/S after 24 h incubation. The supernatant was separated from the pellet by centrifugation and the pellets were then resuspended in methanol and submitted to sonication.

The pH, initially set up at 7, did not show any variations in all media between t = 0 and t = 24 h. As previously mentioned, after 24 h incubation, a change of color in the media inoculated with R-72008 was observed: the supernatants in both rich and poor media were very clear and did not seem to contain the red pigments A/S anymore (Figure 3). This visual observation was confirmed by the study of the HPLC-DAD chromatograms.

**FIGURE 4:**
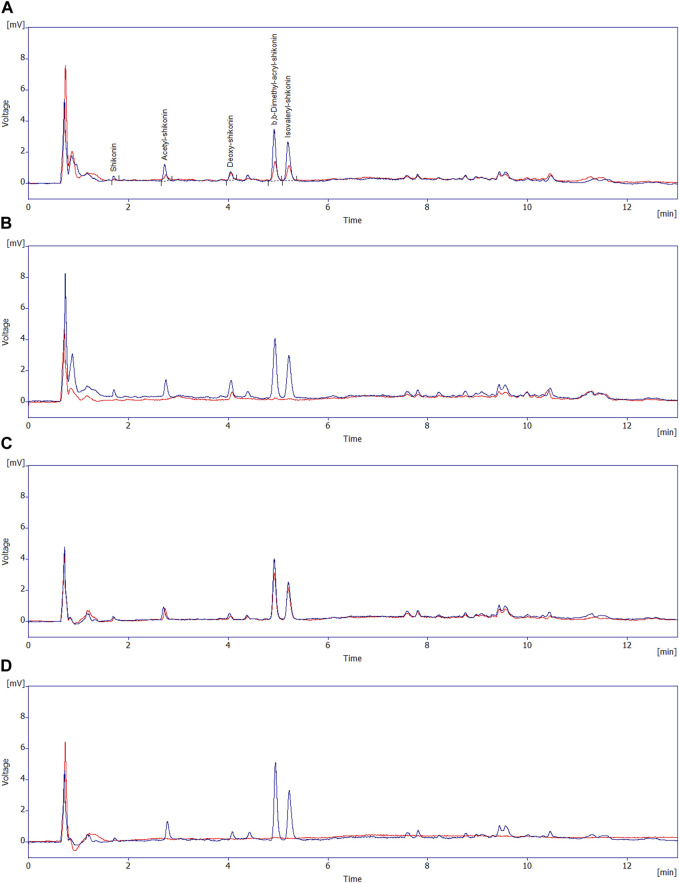
Overlay of the HPLC-DAD (at 520 nm) chromatograms of the supernatants of: **(A)** R2B + A/S medium uninoculated control at t = 0 (blue line) and t = 24 h (red line) and; **(B)** R2B + A/S medium inoculated with *Pseudomonas* sp. R-27008 at t = 0 (blue line) and t = 24 h (red line); **(C)** MSM + A/S medium uninoculated control at t = 0 (blue line) and t = 24 h (red line) and; **(D)** MSM + A/S medium inoculated with *Pseudomonas* sp. R-27008 at t = 0 (blue line) and t = 24 h (red line). The peaks at the retention times 1–6 min correspond to the monomeric A/S initially present in the A/S mixture.

In supernatants of both R2B + A/S and MSM + A/S media inoculated with *Pseudomonas* sp. R-72008 after 24 h incubation, the peaks of alkannin/shikonin, acetyl-alkannin/shikonin, deoxy-shikonin, β,β-dimethylacryl-alkannin/shikonin, and isovaleryl-alkannin/shikonin had disappeared (Figure 4). Moreover, peaks with retention time from 6 min to 12 min, associated with A/S oligomers, had reduced strongly in R2B + A/S inoculated with R-72008 at t= 24 h, and had completely disappeared in the MSM with the bacteria (Figure 4). In the uninoculated controls, the peaks characteristic of monomeric and oligomeric A/S remain present after 24 h incubation in both R2B and MSM media. However, the peaks of β,β-dimethylacryl-alkannin/shikonin (Rt = 4.96 min); and isovaleryl-alkannin/shikonin (Rt = 5.24 min) reduced by 51% and 50%, respectively, in R2B + A/S (Supplementary Table S4), showing an effect of the nutrient medium on the A/S compounds.

The pellet from the nutrient medium R2B + A/S inoculated with *Pseudomonas* sp. R-72008 showed a dark purple color (Figure 3). The HPLC-DAD chromatograms of pellets from the R2B + A/S medium at 0 and 24 h showed that peaks associated with monomeric A/S (Rt < 6 min) did not change much in both inoculated and non-inoculated media and compared to the supernatants (Supplementary Figure S2A,B; Figure 4). An increased intensity of the peaks with retention time > 6 min was observed in the medium inoculated with R-72008 compared to the uninoculated control (Supplementary Figure S2A,B). The peak intensity of R2B + A/S inoculated with R-72008 increased already at 0 h and kept increasing at 24 h.

In the MSM + A/S pellet, reduction of the monomeric peaks was observed for both the uninoculated control and the medium inoculated with R-72008, showing also an effect of the MSM medium on the A/S compounds. No significant variation was observed for the oligomeric compounds in MSM + A/S pellets of both the uninoculated control and the medium inoculated with R-72008 (Supplementary Figure S2C,D).

**FIGURE 5:**
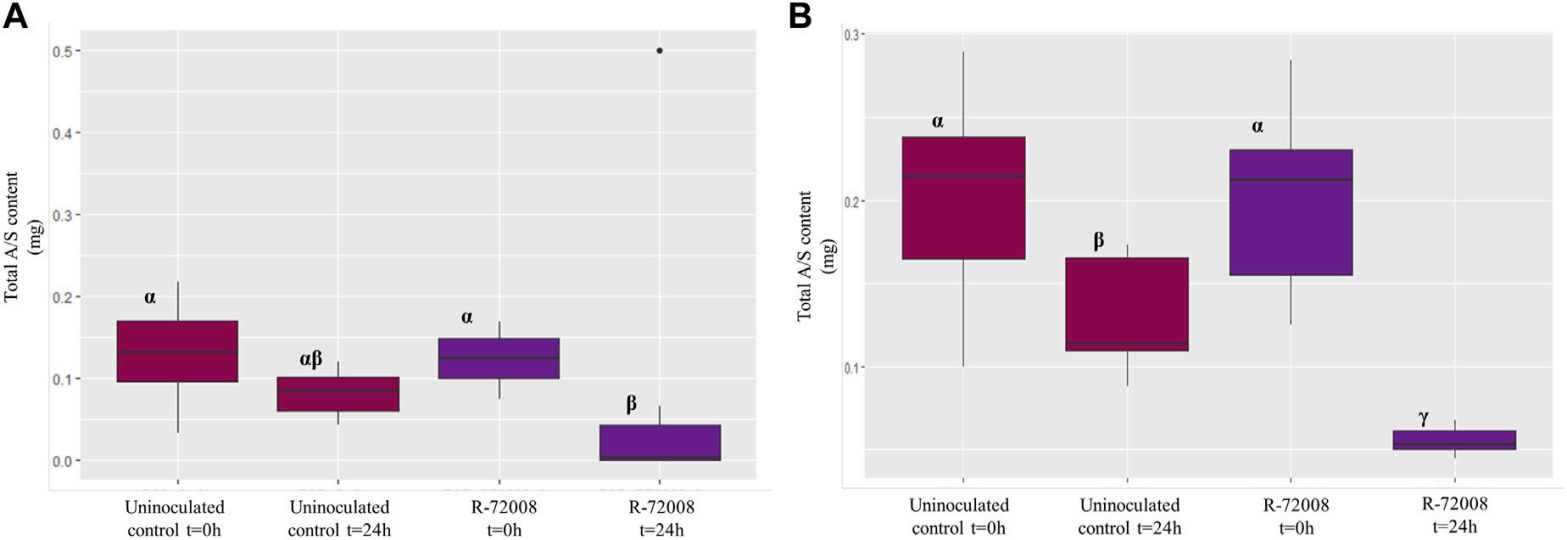
Boxplot representation of the median (horizontal black line), upper and lower quartiles (box), for the total A/S content in the culture media inoculated with *Pseudomonas* sp. R-72008. Panel **(A)** results in R2B + A/S medium (3 biological replicates per treatment). Data were analysed by a Kruskal–Wallis test followed by a Dunn test (*p* < 0.05). Panel **(B)** results in MSM + A/S medium (3 biological replicates per treatment). Data were analysed by a one-factor ANOVA followed by a Newman-Keuls test (*p* < 0.05). Treatments associated with the same letter did not differ significantly.

Statistics were also performed on the total monomeric A/S content of both supernatant and pellet (Supplementary Table S3, S4). For the assay in the R2B medium, the residuals did not follow a normal distribution. The statistical analysis (Kruskal–Wallis) showed that after 24h, R-72008 generated a significant decrease of the initial total monomeric A/S content compared to the medium R2B + A/S inoculated at t = 0 h, but not compared to the uninoculated control at 24 h (Figure 5A, Figure 3, Supplementary Figure S2A,B). For the assay in the MSM medium, the residuals did follow a normal distribution. The one-factor ANOVA followed by a Newman-Keuls test showed that the strain *Pseudomonas* sp. R-72008 generated a significant decrease of the initial total monomeric A/S content compared to the medium MSM inoculated at t = 0 h and compared to the uninoculated control at 24 h (Figure 5B, Figure 3, Supplementary Figure S2C,D). These results confirmed the “medium effect” observed previously in the chromatograms of the supernatant of R2B and the pellet of MSM: a decrease of A/S was observed not only in presence of *Pseudomonas* sp. R-72008, but also in the uninoculated controls. Specifically, after 24 h of incubation, the content of A/S in controls of MSM medium decreased 37.6% and in controls of R2B medium 38.5%.

The bacterium R-72008 seemed therefore to have a strong effect on the production of A/S oligomers in R2B + A/S medium but to not have any effect on the monomeric A/S. In contrast, the presence of *Pseudomonas* sp. R-72008 in MSM + A/S seemed to reduce strongly the amount of monomeric A/S, and rather decrease the amount of A/S oligomers. This confirms our previous observation of R-72008 using A/S as carbon source to support its growth in the MSM medium.

#### 3.2.3 UPLC-ESI-MS/MS analysis

To confirm the presence of oligomers and tentatively identify them, an UPLC-ESI-MS/MS analysis on pellets of *Pseudomonas* sp. R-72008 grown 24 h in R2B medium supplemented with A/S was performed. Identification of the compounds was performed based on LC-MS analyses of A/S standards, exact ionic masses-base peaks (b.p.) and fragmentation patterns, and our previous works (Spyros et al., 2005; Assimopoulou et al., 2008; Assimopoulou et al., 2009; Tappeiner et al., 2014).

**FIGURE 6:**
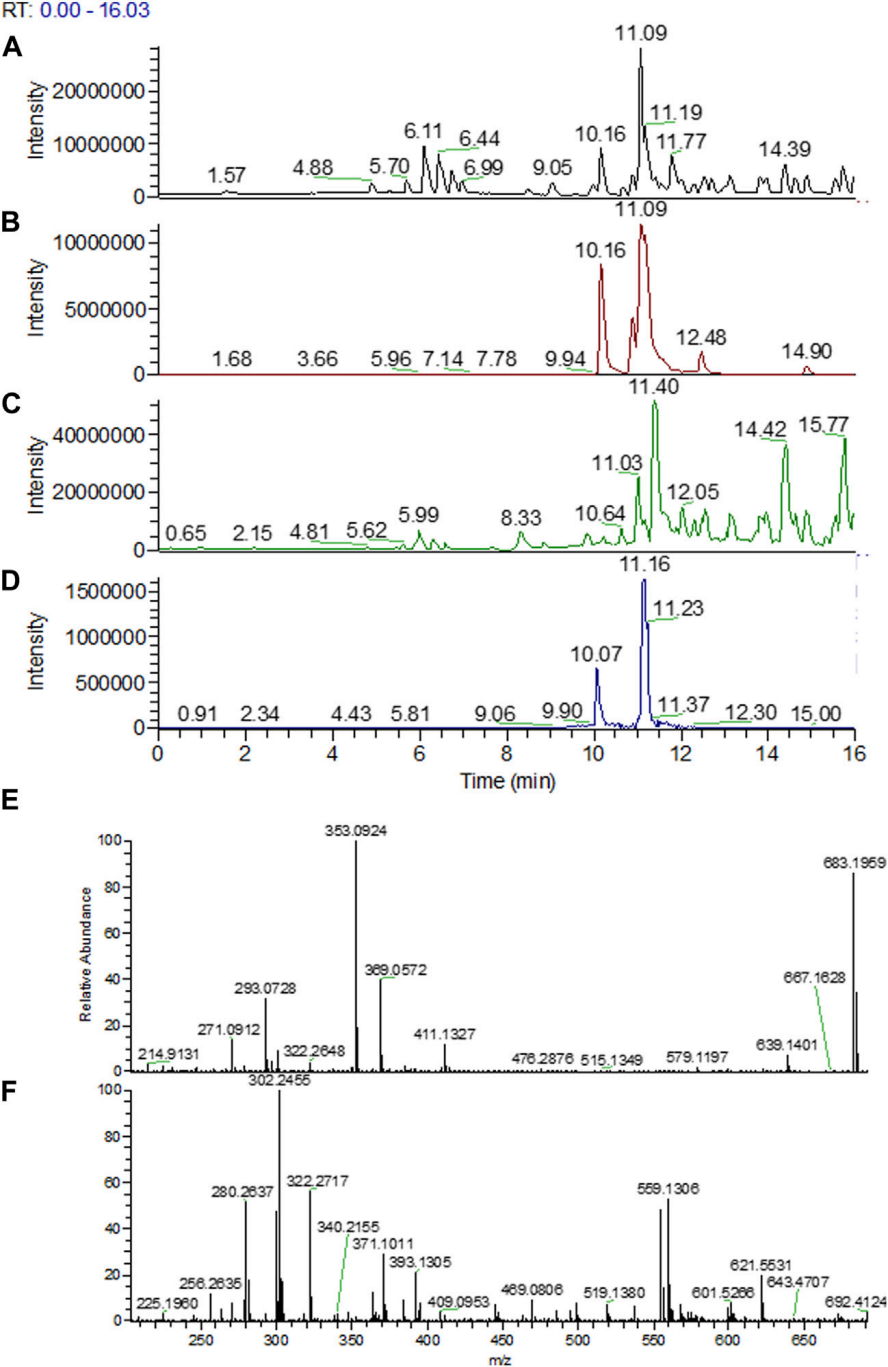
UPLC-ESI-MS/MS chromatograms of methanolic extract of the pellets from R2B medium supplemented with A/S: **(A)** uninoculated control sample at 0 h; base peak; **(B)** uninoculated control sample at 0 h; selected mass ranges (353; 381; 393; 395; 543; 559; 643 &871 m/z); **(C)** *Pseudomonas sp.* R-72008 after 24 h incubation; base peak; **(D)** *Pseudomonas sp.* R-72008 after 24 h incubation; selected mass ranges (353; 381; 393; 395; 543; 559; 643 &871 m/z). Indicative mass spectra at positive ionization of: **(E)** peak at Rt 10.16 min (Chromatogram B) and **(F)** peak at Rt 11.16 min (Chromatogram D).

As shown in Figure 6, in pellets of R2B medium at t = 0 h, monomeric A/S compounds were detected both in the uninoculated control and in the medium with *Pseudomonas* sp. R-72008. Specifically, acetyl-alkannin/shikonin (*m/z* 271.0924 b.p., 353.0942 [M + Na]^+^) at 10.16 min, isobutyl-alkannin/shikonin (*m/z* 271.0919 b.p., 381.1245 [M + Na]^+^) at 10.90 min, β,β-dimethylacryl-alkannin/shikonin (*m/z* 271.0920 b.p., 371.0945 [M + H]^+^, 393.1240 [M + Na]^+^) at 11.05 min and isovaleryl-alkannin/shikonin (*m/z* 271.0921 b.p., 395.1397 [M + Na]^+^) at 11.19 min (Figures 6 A,B). However, none of these monomers were detected in the sample of R2B medium inoculated with *Pseudomonas* sp. R-72008 after 24 h incubation.

Also, at 12.4 min a peak with *m/z* 643.2448 ([M + H]^+^) was detected (Figure 6), which is an indication that isovaleryl-alkannin/shikonin dimers are present in the samples. Furthermore, *m/z* 871.2966 ([M + H]^+^) was perceived at t = 14.7 min indicating that acetyl-alkannin/shikonin trimers are present in the samples. At 11.03 min, a low intensity peak with *m/z* 559.1288 ([M + H]^+^) was detected (Figure 6), corresponding to alkannin/shikonin dimers (Spyros et al., 2005; Assimopoulou et al., 2008; Assimopoulou et al., 2009; Tappeiner et al., 2014). Moreover, at 11.7 min, a compound with 543.0988 m*/z* [M + H]^+^ and 565.0695 [M + Na]^+^ was detected, with 542 being the molecular weight of a compound (Assimopoulou et al., 2008) in such samples (hexane extracts of *A. tinctoria*, shikonin and acetyl-shikonin), but this could not be identified with known structures till date (Figures 6C,D). Finally, after 13 min at the LC-MS chromatograms, all compounds detected have Mw higher than 800 (and chromophore groups similar to A/S as shown in the PDA spectrum of the HPLC-DAD analysis), indicating A/S oligomers.

The UPLC-ESI-MS/MS analysis confirmed the effect of R-72008 on the increase of oligomeric A/S. More specifically, the amount of isovaleryl-alkannin/shikonin dimers, acetyl-alkannin/shikonin trimers, alkannin/shikonin dimers increase after 24 h incubation. Moreover, bigger oligomers with no characterized structure were created (Figure 6).

## 4 Discussion

A/S are plant metabolites with various medicinal effects. With the emergence of microbial resistance to antibiotics, studying such molecules and their interactions with bacteria is key to design new efficient antibiotics. In a previous study of our group (Rat et al., 2021), endophytic bacteria were isolated from the roots of wild-growing *Alkanna tinctoria* (L.) Tausch, a plant producing A/S, and Gram-negative bacteria were shown to be resistant to the antimicrobial properties of these compounds. In this work, we aimed to study the response of resistant bacteria to A/S.

In a preliminary screening in R2B medium supplemented with A/S, a change of the medium color from pink to purple was observed for most of the bacterial treatments and was correlated with the appearance of a peak at 410 nm in spectrophotometric analysis (Figure 2, Supplementary Table S2). In previous works, the purple coloration of A/S compounds has commonly been attributed to an alkalinization of the medium (Papageorgiou et al., 1999; Tatsumi et al., 2016) or A/S polymerization (A. Assimopoulou, unpublished data). In this study, the pH did not change during incubation with bacteria, and the HPLC-DAD and UPLC-ESI-MS/MS analyses revealed that the 407 nm wavelength was correlated with absorbance of A/S oligomers. We observed indeed that monomeric A/S disappeared from the supernatant while they were still present in the uninoculated controls, confirming that monomeric A/S were likely converted into A/S oligomers in the presence of *Pseudomonas* strain R-72008. Oligomerization of A/S by bacteria was also reported in the study of Meselhy et al. (1994). They demonstrated that the anaerobic incubation of shikonin with *Bacteroides fragillis* led to the formation of shikonin dimers called shikometabolins. Interestingly, this bacterial effect on A/S was not specific to bacteria isolated from A/S-producing plants, as we found several reference strains from the BCCM/LMG Bacteria Collection isolated from different sources expressed a similar behavior. Therefore, the formation of colored oligomers seems to depend on a bacterial mechanism shared by many bacteria.

The high production of A/S oligomers was only observed in the nutrient R2B + A/S medium and could be noticed from t = 0. At t = 0, active bacterial metabolism does not have time to adjust to new growth conditions and it is unlikely that bacteria are actively converting monomeric A/S into oligomers. Naphthoquinones such as A/S have two modes of action: oxidative and electrophilic. In a previous study, Assimopoulou et al. (2008) showed the possible polymerization pattern of A/S. The process of polymerization involves initially each of the carbon C-6 or C-7 of the A/S monomer acting as electrophile with the side chain hydroxyl group of another monomer. The electron(s) of the quinone radicals can then couple in various ways and thus the initial dimerization can occur at different positions, and the polymerization can continue further and thus other alkannin/shikonin oligomers might be produced. Due to the complexity of the A/S molecule, where both electron donor and acceptor reactions take place, the mechanism of polymerization may occur in several ways, depending on the conditions applied each time. Polymerization of alkannin/shikonin in alkaline media and during hydrolysis of their esters proceeds via coupling between the semiquinone forms and phenoxyl radicals of each of the phenolic hydroxyl groups; several possible dimer formulae have been proposed (Assimopoulou and Papageorgiou, 2004). Dimers with less steric hindrance will predominate. Such observations would explain the different shades of color found during the bacterial screening.

A/S are dyes, able to color the bacteria, and so to adhere to the bacterial membrane. Also, our protocol of metabolite extraction involved sonication which opens bacterial cells, releasing their contents. Protons needed for the formation of electrophiles can come from the bacterial metabolism, released after sonication. Such indirect processes would explain the presence of A/S oligomers immediately after addition of the bacteria, and the increase of A/S oligomers in parallel with the bacterial growth in the R2B + A/S medium. Isolation - at a large scale - of the compounds detected by LC-MS/MS and further LC-MS and NMR analysis could help to elucidate which type of product is produced when bacteria are present, and thus might provide a better insight in what chemical reaction/transformation occurred. This requires scale up of the experiment to isolate and purify compounds.

In MSM + A/S medium, no effect on A/S oligomer production could be noticed. However, a strong decrease of monomeric A/S was observed in the medium inoculated with R-72008 compared to the uninoculated control. Also, the bacterium was not able to grow in MSM in absence of supplement but could grow as much in MSM + A/S than in MSM + glucose. These results indicate that R-72008 was able to use A/S to support its growth. R-72008 did not grow more in the nutrient medium R2B + A/S compared to the MSM + A/S, despite the availability of a wider source of nutrients. Such behavior raises the hypothesis of a mechanism of catabolite repression. We suggest that in presence of more favorable carbon sources, the production of enzymes able to metabolize A/S will be inhibited. To our knowledge, no mechanism of naphthoquinone metabolization has been described in bacteria. So far, only the metabolization of the naphthoquinone lawsone has been observed, without identification of the genes/enzymes involved (Yang et al., 2017). Genes encoding enzymes responsible for the metabolism of naphthalene compounds, which have a similar ring structure to naphthoquinone, were described in several bacteria. *Pseudomonas putida* CSV86, was shown to detoxify and metabolize 1-methylnaphthalene to use it as sole source of carbon and energy (Basu et al., 2003). Moreover, the nah-1 operon and the sgp operon have been shown to be involved in naphthalene degradation by *Pseudomonas putida* AL5 (Izmalkova et al., 2013). Specific genes coding for enzymes metabolizing naphthoquinones might exist in strain R-72008, but no genes coding for naphthoquinone utilization have been annotated in bacteria yet. In the future, such genes could be searched for through the production of bacterial mutants.

This study showed for the first time that a bacterium, *Pseudomonas* sp. R-72008, is able to use naphthoquinones, especially A/S, as sole carbon source. We hypothesized that this metabolism involves a mechanism of catabolite repression. The bacterial response to A/S (oligomerization, utilization) is complex and likely involves several mechanisms from bacterial electric charges to specific enzymatic reactions. Although these findings should be validated through mutant and proteomics analysis, they are promising for the elucidation of a new bacterial metabolic capacities. Especially, the dual redox nature of naphthoquinones makes them a promising approach against microbial pathogens (Mone et al., 2021). Understanding the mechanism of naphthoquinone metabolization would be crucial to design effective antibiotics. Moreover, R-72008 is able to use A/S for its growth and the ability to produce such enzymes is likely linked with the resistance of the bacteria against these plant antimicrobials. Such activity might provide a competitive advantage to colonize A/S-producing plants. This should be validated through colonization assays *in planta* with mutants deficient in A/S-utilization enzymes. Also, as A/S are specific secondary metabolites produced by members of the Boraginaceae family such as *Lithospermum* spp., *Alkanna* spp., *Arnebia* spp., *Onosma* spp. and *Echium* spp., testing the ability to use A/S in *Pseudomonas* strains isolated from other sources than A/S-producing plants can open-up the perspective of studying the evolutionary process behind these bacterial properties.

## Data Availability

The original contributions presented in the study are included in the article/Supplementary Material, further inquiries can be directed to the corresponding author.
