# Supplementary material for: Bacterial responses to plant antimicrobials: the case of alkannin and shikonin derivatives
Source: Front Pharmacol. 2023 Aug 7;14:1244270. doi: 10.3389/fphar.2023.1244270 (PMC10440953; doi:10.3389/fphar.2023.1244270)
Supplement: Supplementary file 1 [file DataSheet1.docx]

## **Supplementary materials**

Supplementary Table S1: Effect of bacteria on the A/S mixture. Change of medium colour and absorbance at 410 nm and 520 nm were recorded. The relative intensity of the 410 nm is indicated as “+” or “++”; ‘-’ indicates no peak at 410 nm.

| **Strain** | **Identification** | **Medium color** | **Peak at 410 nm** | | **Peak at 520 nm** | **Strain** | **Identification** | **Medium color** | **Peak at 410 nm** | | | **Peak at 520 nm** |
| --- | --- | --- | --- | --- | --- | --- | --- | --- | --- | --- | --- | --- |
| Control |  | Pinkish | 0.734 | - | 0.992 | R-74587 | *Pedobacter* sp. | Light pink | | 1.062 | + | 1.063 |
| R-71825 | *Roseomonas* sp. | Pinkish as control | 0.706 | - | 0.646 | LMG 2210 | *Pseudomonas marginalis pv. marginalis* | Dark purple | | 1.819 | ++ | 1.432 |
| R-71838 | *Pseudomonas* sp. | Dark purple | 1.710 | ++ | 1.158 | LMG 2152 | *Pseudomonas caricapapayae* | Dark purple | | 1.070 | + | 0.968 |
| R-71842 | *Pseudomonas* sp. | Dark purple | 1.846 | ++ | 1.281 | LMG 2352 | *Pseudomonas viridiflava* | Dark purple | | 1.416 | + | 1.083 |
| R-71971 | *Brevibacillus* sp. | Pinkish as control | 0.829 | - | 0.743 | LMG 5060 | *Pseudomonas coronafaciens* | Dark purple | | 1.128 | + | 1.065 |
| R-71976 | *Pseudomonas* sp*.* | Dark purple | 1.441 | ++ | 1.092 | LMG 5090 | *Pseudomonas syringae pv. tagetis* | Dark purple | | 1.575 | + | 1.306 |
| R-72008 | *Pseudomonas* sp. | Light purple | 0.799 | + | 0.599 | LMG 5093 | *Pseudomonas syringae pv. tomato* | Light purple | | 1.111 | + | 1.090 |
| R-72015 | *Pantoea* sp. | Light purple | 1.008 | + | 0.823 | LMG 5694 | *Pseudomonas amygdali* | Light purple | | 0.518 | + | 0.641 |
| R-72016 | *Variovorax* sp. | Dark purple | 0.586 | - | 0.651 | LMG 13184 | *Pseudomonas amygdali* | Light brown | | 1.194 | + | 0.944 |
| R-72066 | *Rhizobium* sp. | Very Light pink | 0.884 | + | 0.613 | LMG 21995 | *Pseudomonas lurida* | Dark purple | | 1.987 | ++ | 1.358 |
| R-72074 | *Pseudomonas* sp. | Dark purple | 1.764 | ++ | 1.131 | LMG 23076 | *Pseudomonas palleroniana* | Dark purple | | 2.103 | ++ | 1.513 |
| R-72102 | *Pseudomonas* sp. | Dark purple | 1.992 | ++ | 1.401 | LMG 26839 | *Pseudomonas punonensis* | Dark purple | | 1.402 | + | 1.093 |
| R-72160 | *Rhizobium* sp. | Dark purple | 1.460 | + | 1.041 | LMG 26898 | *Pseudomonas asturiensis* | Dark purple | | 1.239 | + | 1.115 |
| R-72164 | *Pseudomonas* sp. | Dark purple | 1.805 | + | 1.091 | LMG 27394 | *Pseudomonas guariconensis* | Dark purple | | 1.633 | ++ | 0.991 |
| R-72249 | *Pedobacter* sp*.* | Pinkish as control | 0.457 | - | 0.669 | LMG 27930 | *Pseudomonas karstica* | Same as control | | 0.438 | - | 0.517 |
| R-72395 | *Massilia* sp. | Light pink | 0.907 | + | 1.073 | LMG 28435 | *Pseudomonas lactis* | Light brown | | 1.176 | ++ | 0.779 |
| R-72406 | *Stenotrophomonas* sp. | Light purple | 0.879 | + | 0.861 | LMG 28439 | *Pseudomonas paralactis* | Light brown | | 1.122 | - | 0.867 |
| R-72433 | *Rhizobium* sp. | Light pink | 0.620 | - | 0.664 | LMG 28456 | *Pseudomonas endophytica* | Dark purple | | 1.548 | + | 1.250 |
| R-72446 | *Variovorax* sp. | Dark purple | 0.640 | - | 0.478 | LMG 28495 | *Pseudomonas syringae* pv. *porri* | Dark pink | | 1.186 | + | 1.030 |
| R-72498 | *Pantoea* sp*.* | Light purple | 0.699 | + | 0.597 | LMG 28558 | *Pseudomonas coleopterorum.* | Light purple | | 1.055 | + | 0.939 |
| R-72599 | *Pseudomonas* sp. | Dark purple | 1.502 | + | 1.103 | LMG 30013 | *Pseudomonas floridensis* | Dark purple | | 1.015 | ++ | 0.743 |
| R-73080 | *Roseomonas* sp. | Dark purple | 1.063 | - | 1.161 | LMG 30275 | *Pseudomonas glycinae* | Dark purple | | 2.328 | ++ | 1.660 |
| R-73098 | *Xanthomonas* sp. | Light pink | 0.681 | + | 0.585 | LMG 30830 | *Pseudomonas bubulae* | Lighter pink than control | | 1.229 | + | 1.108 |
| R-73111 | *Phyllobacterium* sp. | Pinkish as control | 0.676 | + | 0.832 | LMG 30182 | *Pseudomonas bohemica* | Light purple | | 0.831 | + | 0.728 |
| R-74283 | *Stenotrophomonas* sp. | Dark purple | 1.063 | + | 1.063 | LMG 31089 | *Pseudomonas kirkiae* | Dark purple | | 1.322 | + | 1.148 |

Supplementary Table S2: Bacterial growth recorded at 590 nm in R2B medium supplemented with 250 mg/L lawsone. The values at least two times higher than the control are indicated as “+”.

| **Strain** | **Identification** | **Growth** | **Strain** | **Identification** | **Growth** |
| --- | --- | --- | --- | --- | --- |
| Control |  |  | R-74587 | *Pedobacter* sp. | + |
| R-71825 | *Roseomonas* sp. | - | LMG 2210 | *Pseudomonas marginalis* pv. *marginalis* | + |
| R-71838 | *Pseudomonas* sp. | + | LMG 2152 | *Pseudomonas caricapapayae* | + |
| R-71842 | *Pseudomonas* sp. | + | LMG 2352 | *Pseudomonas viridiflava* | + |
| R-71971 | *Brevibacillus* sp. | - | LMG 5060 | *Pseudomonas coronafaciens* | + |
| R-71976 | *Pseudomonas* sp*.* | + | LMG 5090 | *Pseudomonas syringae pv. tagetis* | + |
| R-72008 | *Pseudomonas* sp. | + | LMG 5093 | *Pseudomonas syringae pv. tomato* | + |
| R-72015 | *Pantoea* sp. | + | LMG 5694 | *Pseudomonas amygdali* | + |
| R-72016 | *Variovorax* sp. | + | LMG 13184 | *Pseudomonas amygdali* | + |
| R-72066 | *Rhizobium* sp. | + | LMG 21995 | *Pseudomonas lurida* | + |
| R-72074 | *Pseudomonas* sp. | + | LMG 23076 | *Pseudomonas palleroniana* | + |
| R-72102 | *Pseudomonas* sp. | + | LMG 26839 | *Pseudomonas punonensis* | + |
| R-72160 | *Rhizobium* sp. | + | LMG 26898 | *Pseudomonas asturiensis* | + |
| R-72164 | *Pseudomonas* sp. | + | LMG 27394 | *Pseudomonas guariconensis* | + |
| R-72249 | *Pedobacter* sp. | + | LMG 27930 | *Pseudomonas karstica* | - |
| R-72395 | *Massilia* sp. | + | LMG 28435 | *Pseudomonas lactis* | + |
| R-72406 | *Stenotrophomonas* sp*.* | + | LMG 28439 | *Pseudomonas paralactis* | - |
| R-72433 | *Rhizobium* sp. | + | LMG 28456 | *Pseudomonas endophytica* | + |
| R-72446 | *Variovorax* sp. | - | LMG 28495 | *Pseudomonas syringae pv. porri* | + |
| R-72498 | *Pantoea* sp. | + | LMG 28558 | *Pseudomonas coleopterorum* | + |
| R-72599 | *Pseudomonas* sp. | + | LMG 30013 | *Pseudomonas floridensis* | + |
| R-73080 | *Roseomonas* sp. | - | LMG 30275 | *Pseudomonas glycinae* | + |
| R-73098 | *Xanthomonas* sp. | + | LMG 30830 | *Pseudomonas bubulae* | + |
| R-73111 | *Phyllobacterium* sp. | - | LMG 30182 | *Pseudomonas bohemica* | + |
| R-74283 | *Stenotrophomonas* sp. | + | LMG 31089 | *Pseudomonas kirkiae* | + |

Table S3: Content of monomers of alkannin/shikonin (A/S), acetyl-alkannin/shikonin, deoxy- shikonin, β,β-dimethylacryl-alkannin/shikonin and isovaleryl-alkannin/shikonin in MSM medium obtained by HPLC-DAD. Data are expressed in milligrams for the uninoculated control samples and for samples inoculated with Pseudomonas sp. R-72008. The A/S content was measured in supernatant (SN) and bacterial pellet (P). In the sample names B1, B2 and B3 indicate the biological replicates, T1, T2, T3 indicate the technical replicates and t= 0 h and t= 24 h the sampling time. ‘–’ indicates none detected.

| Sample | A/S  (mg) | | Acetyl-A/S  (mg) | | Deoxy-A/S (mg) | | β,β-Dimethyl-A/S (mg) | | Isovaleryl-A/S (mg) | | Total A/S  (mg) |
| --- | --- | --- | --- | --- | --- | --- | --- | --- | --- | --- | --- |
|  | P | SN | P | SN | P | SN | P | SN | P | SN | P+SN |
| Control samples | | | | | | | | | | |  |
| B1T1_0h | 0.0016 | - | 0.0324 | 0.0080 | 0.0027 | - | 0.0571 | 0.0380 | 0.0565 | 0.0180 | 0.2124 |
| B1T2_0h | 0.0020 | - | 0.0533 | 0.0070 | 0.0031 | - | 0.0870 | 0.0300 | 0.0925 | 0.0140 | 0.2889 |
| B1T3_0h | 0.0013 | - | 0.0224 | 0.0090 | - | - | 0.0394 | 0.0600 | 0.0385 | 0.0280 | 0.1986 |
| B2T1_0h | 0.0014 | - | 0.0055 | 0.0080 | 0.0051 | - | 0.0147 | 0.038 | 0.0097 | 0.0170 | 0.0994 |
| B2T2_0h | 0.0020 | - | 0.0308 | - | 0.0038 | - | 0.0606 | 0.0120 | 0.0538 | - | 0.1630 |
| B2T3_0h | 0.0019 | - | 0.0297 | - | 0.0034 | - | 0.0587 | 0.0190 | 0.0521 | - | 0.1648 |
| B3T1_0h | 0.0019 | - | 0.0368 | 0.0090 | 0.0032 | - | 0.0683 | 0.0370 | 0.0647 | 0.0170 | 0.2379 |
| B3T2_0h | 0.0016 | - | 0.0299 | 0.0100 | 0.0021 | - | 0.0517 | 0.0530 | 0.0504 | 0.0210 | 0.2197 |
| B3T3_0h | 0.0019 | - | 0.0451 | 0.0120 | 0.0026 | - | 0.0773 | 0.0470 | 0.0769 | 0.0210 | 0.2838 |
| B1T1_24h | 0.0010 | - | 0.0112 | 0.0070 | - | - | 0.0259 | 0.0300 | 0.0220 | 0.0160 | 0.1131 |
| B1T2_24h | 0.0012 | 0.0060 | 0.0165 | 0.0090 | 0.0030 | - | 0.0351 | 0.0180 | 0.0308 | 0.0100 | 0.1296 |
| B1T3_24h | 0.0010 | 0.0060 | 0.0117 | - | 0.0024 | - | 0.0257 | 0.0240 | 0.0218 | 0.0120 | 0.1046 |
| B2T1_24h | 0.0013 | - | 0.0229 | 0.0070 | 0.0027 | - | 0.0472 | 0.0260 | 0.0435 | 0.0150 | 0.1656 |
| B2T2_24h | 0.0016 | - | 0.0255 | 0.0080 | 0.0035 | - | 0.0540 | 0.0210 | 0.0486 | 0.0110 | 0.1732 |
| B2T3_24h | 0.0014 | - | 0.0184 | - | 0.0034 | - | 0.0398 | 0.0170 | 0.0339 | - | 0.1139 |
| B3T1_24h | 0.0013 | - | 0.0193 | 0.0100 | 0.0029 | - | 0.0400 | 0.0410 | 0.0346 | 0.0210 | 0.1701 |
| B3T2_24h | 0.0009 | - | 0.0015 | - | 0.0031 | - | 0.0054 | 0.0530 | 0.0033 | 0.0210 | 0.0882 |
| B3T3_24h | 0.0012 | - | 0.0067 | - | 0.0039 | - | 0.0172 | 0.0470 | 0.0122 | 0.0210 | 0.1092 |
| Samples inoculated with strain R-72008 | | | | | | | | | | |  |
| B1T1_0h | 0.0014 | - | 0.0299 | 0.0100 | 0.0024 | - | 0.0534 | 0.0470 | 0.0535 | 0.0240 | 0.2216 |
| B1T2_0h | 0.0017 | - | 0.0371 | 0.0080 | 0.0029 | - | 0.0638 | 0.0340 | 0.0648 | 0.0180 | 0.2303 |
| B1T3_0h | 0.0013 | - | 0.0185 | 0.0090 | 0.0023 | - | 0.0333 | 0.0390 | 0.0304 | 0.0210 | 0.1548 |
| B2T1_0h | 0.0019 | - | 0.0410 | 0.0090 | 0.0032 | - | 0.0705 | 0.0370 | 0.0719 | 0.0180 | 0.2525 |
| B2T2_0h | 0.0019 | - | 0.0523 | 0.0070 | 0.0029 | - | 0.0838 | 0.0310 | 0.0894 | 0.0160 | 0.2843 |
| B2T3_0h | 0.0015 | - | 0.0266 | 0.0100 | 0.0024 | - | 0.0486 | 0.0500 | 0.0468 | 0.0260 | 0.2119 |
| B3T1_0h | 0.0017 | - | 0.0232 | - | 0.0034 | - | 0.0455 | 0.0100 | 0.0411 | - | 0.1249 |
| B3T2_0h | 0.0016 | - | 0.0238 | 0.0080 | 0.0030 | - | 0.0458 | 0.0380 | 0.0422 | 0.0180 | 0.1804 |
| B3T3_0h | 0.0015 | - | 0.0242 | - | 0.0027 | - | 0.0451 | 0.0180 | 0.0427 | 0.0090 | 0.1432 |
| B1T1_24h | - | - | 0.0070 | - | - | - | 0.0312 | - | 0.0291 | - | 0.0673 |
| B1T2_24h | - | - | 0.0052 | - | - | - | 0.0243 | - | 0.0209 | - | 0.0504 |
| B1T3_24h | - | - | 0.0050 | - | - | - | 0.0244 | - | 0.0206 | - | 0.05 |
| B2T1_24h | - | - | 0.0084 | - | - | - | 0.0282 | - | 0.0244 | - | 0.0611 |
| B2T2_24h | - | - | 0.0064 | - | - | - | 0.0214 | - | 0.0171 | - | 0.0449 |
| B2T3_24h | - | - | 0.0075 | - | - | - | 0.0247 | - | 0.0207 | - | 0.0529 |
| B3T1_24h | - | - | 0.0086 | - | - | - | 0.0298 | - | 0.0270 | - | 0.0654 |
| B3T2_24h | - | - | 0.0063 | - | - | - | 0.0229 | - | 0.0192 | - | 0.0484 |
| B3T3_24h | - | - | 0.0079 | - | - | - | 0.0271 | - | 0.0239 | - | 0.0589 |

Table S4: Content of monomers of alkannin/shikonin (A/S), acetyl-alkannin/shikonin, deoxy-shikonin, β,β-dimethylacryl-alkannin/shikonin and isovaleryl-alkannin/shikonin in R2B +A/S medium obtained by HPLC-DAD. Data are expressed in milligrams for the uninoculated control samples and for the samples inoculated with Pseudomonas sp. R-72008. The A/S content was measured in supernatant (SN) and bacterial pellet (P). In the sample names B1, B2 and B3 indicate the biological replicates, T1, T2, T3 indicate the technical replicates and t= 0 h and t= 24 h the sampling time. ‘-’ indicates none detected.

| Sample | A/S  (mg) | | Acetyl-A/S  (mg) | | Deoxy-A/S  (mg) | | β,β-Dimethylacryl-A/S (mg) | | Isovaleryl-A/S (mg) | | Total A/S  (mg) |
| --- | --- | --- | --- | --- | --- | --- | --- | --- | --- | --- | --- |
|  | P | SN | P | SN | P | SN | P | SN | P | SN | P+SN |
| Control samples | | | | | | | | | | | |
| B1T1_0h | - | - | 0.0058 | 0.0100 | - | - | 0.0105 | 0.0330 | 0.0114 | 0.0190 | 0.0897 |
| B1T2_0h | - | - | 0.0074 | 0.009 | - | - | 0.0138 | 0.0310 | 0.0150 | 0.0190 | 0.0952 |
| B1T3_0h | - | - | 0.0017 | - | - | - | 0.0037 | 0.0150 | 0.0035 | 0.0080 | 0.0319 |
| B2T1_0h | - | - | 0.0037 | 0.0180 | - | 0.0060 | 0.0072 | 0.0770 | 0.0076 | 0.0440 | 0.1635 |
| B2T2_0h | - | - | 0.0101 | 0.0160 | - | 0.0060 | 0.0164 | 0.0680 | 0.0173 | 0.0380 | 0.1718 |
| B2T3_0h | - | - | 0.0094 | 0.0070 | - | - | 0.0172 | 0.0310 | 0.0177 | 0.0150 | 0.0973 |
| B3T1_0h | - | - | 0.0071 | 0.0140 | - | 0.0050 | 0.0120 | 0.0500 | 0.0132 | 0.0300 | 0.1313 |
| B3T2_0h | - | - | 0.0041 | 0.0190 | - | 0.0060 | 0.0094 | 0.0780 | 0.0084 | 0.0450 | 0.1699 |
| B3T3_0h | - | - | 0.0105 | 0.0210 | - | 0.0060 | 0.0181 | 0.0890 | 0.0179 | 0.0540 | 0.2165 |
| B1T1_24h | - | - | 0.0031 | - | - | - | 0.0131 | 0.0170 | 0.0123 | 0.0100 | 0.0555 |
| B1T2_24h | - | - | 0.0000 | - | - | - | 0.0047 | 0.0210 | 0.0039 | 0.0130 | 0.0426 |
| B1T3_24h | - | - | 0.0045 | - | - | - | 0.0152 | 0.0150 | 0.0152 | 0.0100 | 0.0599 |
| B2T1_24h | - | - | 0.0061 | - | - | - | 0.0177 | 0.0280 | 0.0173 | 0.0120 | 0.0811 |
| B2T2_24h | - | - | 0.0064 | 0.0080 | - | - | 0.0174 | 0.0280 | 0.0169 | 0.0170 | 0.0937 |
| B2T3_24h | - | - | 0.0049 | 0.0090 | - | - | 0.0139 | 0.0370 | 0.0136 | 0.0230 | 0.1014 |
| B3T1_24h | - | - | 0.0086 | - | - | - | 0.0208 | 0.0220 | 0.0208 | 0.0110 | 0.0832 |
| B3T2_24h | - | - | 0.0102 | 0.0090 | 0.0022 | - | 0.0238 | 0.0310 | 0.0240 | 0.0190 | 0.1192 |
| B3T3_24h | - | - | 0.0089 | 0.0110 | 0.0021 | - | 0.0219 | 0.0340 | 0.0212 | 0.0200 | 0.1191 |
| Samples inoculated with strain R72008 | | | | | | | | | | | |
| B1T1_0h | - | - | 0.0028 | 0.0100 | - | 0.0060 | 0.0073 | 0.0430 | 0.0061 | 0.0220 | 0.0972 |
| B1T2_0h | - | - | 0.0023 | 0.0090 | - | - | 0.0057 | 0.0340 | 0.0052 | 0.0180 | 0.0742 |
| B1T3_0h | - | - | 0.0028 | 0.0110 | - | 0.0060 | 0.0060 | 0.0450 | 0.0057 | 0.0230 | 0.0995 |
| B2T1_0h | - | - | 0.0073 | 0.0190 | - | - | 0.0131 | 0.0710 | 0.0143 | 0.0430 | 0.1677 |
| B2T2_0h | - | - | 0.0041 | 0.0190 | - | - | 0.0091 | 0.0720 | 0.0083 | 0.0430 | 0.1555 |
| B2T3_0h | - | - | 0.0068 | 0.0160 | - | - | 0.0145 | 0.0620 | 0.0141 | 0.0350 | 0.1484 |
| B3T1_0h | - | - | 0.0090 | 0.0100 | - | 0.0060 | 0.0161 | 0.0450 | 0.0163 | 0.0220 | 0.1244 |
| B3T2_0h | - | - | 0.0015 | 0.0210 | - | - | 0.0038 | 0.0540 | 0.0033 | 0.0500 | 0.1336 |
| B3T3_0h | - | - | 0.0038 | 0.0130 | - | 0.0060 | 0.0083 | 0.0530 | 0.0078 | 0.0280 | 0.1199 |
| B1T1_24h | - | - | - | - | - | - | - | - | - | - | - |
| B1T2_24h | - | - | - | - | - | - | - | - | - | - | - |
| B1T3_24h | - | - | 0.0026 | - | 0.0058 | - | 0.0308 | - | 0.0258 | - | 0.065 |
| B2T1_24h | - | - | - | - | 0.0022 | - | - | - | - | - | 0.0022 |
| B2T2_24h | - | - | - | - | 0.0028 | - | 0.0072 | - | 0.0052 | - | 0.0152 |
| B2T3_24h | - | - | - | - | 0.0069 | - | 0.0193 | - | 0.0153 | - | 0.0415 |
| B3T1_24h | 0.0011 | - | 0.0313 | - | 0.0206 | - | 0.2240 | - | 0.2227 | - | 0.4997 |
| B3T2_24h | - | - | - | - | - | - | - | - | - | - | - |
| B3T3_24h | - | - | - | - | - | - | - | - | - | - | - |

The standard deviation of the measurements of the technical replicates (T1,T2,T3) in pellet samples in both mediums, was estimated for acetyl-shikonin between ±0.0004 to ±0.0105, for deoxy-shikonin between ±0.0003 to ±0.0021, for β,β-dimethyl-acryl-shikonin between ±0.0003 to ±0.0212 and for isovaleryl-shikonin from ±0.0003 to ±0.0225. Respectively, for the supernatant samples, the SD for acetyl-shikonin was between ±0.0008 to ±0.0048, for deoxy-shikonin ±0.0005, for β,β-dimethyl-acryl-shikonin between ±0.0025 to ±0.0199 and for isovaleryl-shikonin from ±0.0014 to ±0.0125.

Supplementary Table S5: Calibration curves, correlation coefficients, limits of detection (LOD) and quantification (LOQ) for compounds.

| **Compound** | **Calibration Curve (Y=concentration, ppm)** | **R^2^** | **LOD (ppm)** | **LOQ (ppm)** |
| --- | --- | --- | --- | --- |
| Shikonin | 0.211 × (Peak Area) + 0.345 | 0.9996 | 1.05 | 3.20 |
| Acetyl-shikonin | 0.300 × (Peak Area) + 0.682 | 0.9993 | 1.45 | 4.39 |
| Deoxy-shikonin | 0.159 × (Peak Area) + 0.279 | 0.9996 | 1.10 | 3.34 |
| β,β-Dimethylacryl-shikonin | 0.437 × (Peak Area) + 0.556 | 0.9993 | 1.46 | 4.44 |
| Isovaleryl-shikonin | 0.252 × (Peak Area)+ 0.616 | 0.9989 | 1.85 | 5.61 |

(a)


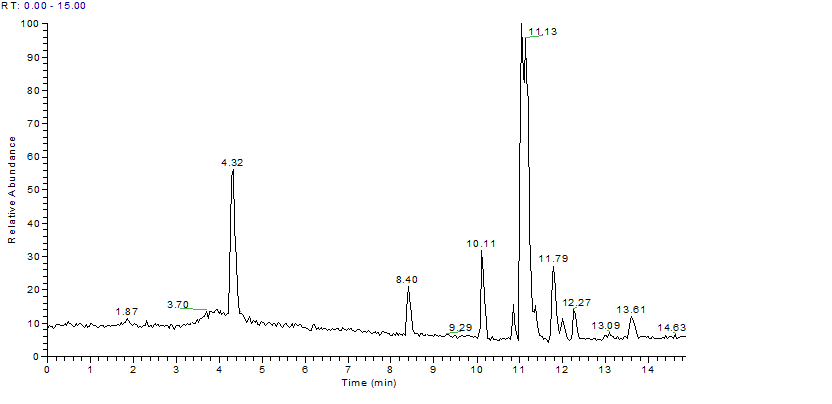


(b)

Supplementary Figure S1: **a)** LC-DAD Chromatogram at 520 nm of the mixture of A/S pigments which used in the degradation study (mobile phase A: water and B: acetonitrile + 0.1% formic acid). Identified peaks: alkannin/shikonin (Rt=1.73 min); acetyl-alkannin/shikonin (Rt=2.79 min); deoxyshikonin (Rt=4.08 min); β,β-dimethylacryl-alkannin/shikonin (Rt=4.96 min); and isovaleryl-alkannin/shikonin (Rt=5.24 min). **b)** LC-ESI-MS of the mixture of pigments (mobile phase A: methanol + 0.1% formic acid and B: water): alkannin/shikonin (Rt= 9.51 min); acetyl-alkannin/shikonin (Rt=10.11 min); isobutyl-alkannin/shikonin (Rt=10.85 min); β,β-dimethylacryl-alkannin/shikonin (Rt= 11.03 min) and isovaleryl-alkannin/shikonin (Rt=11.13 min).


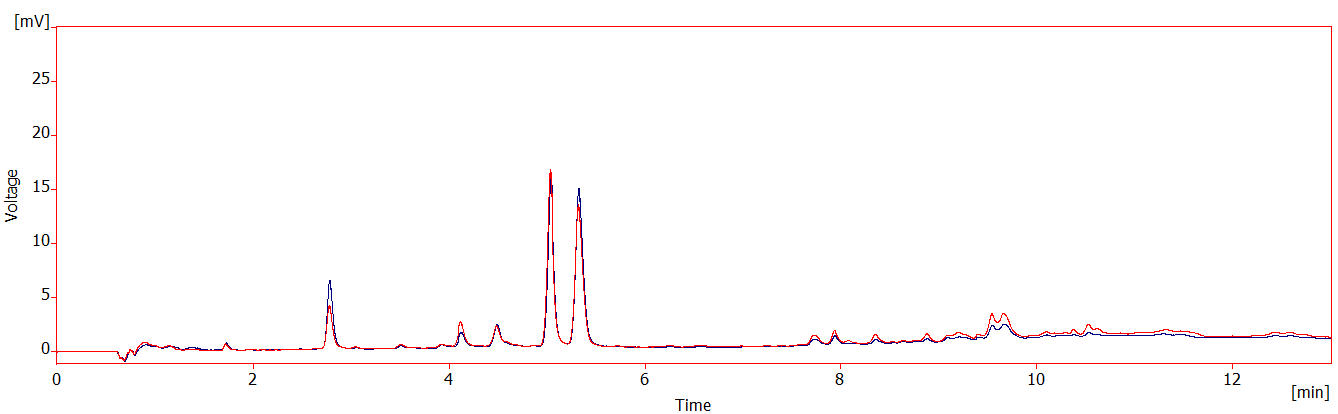


(a)


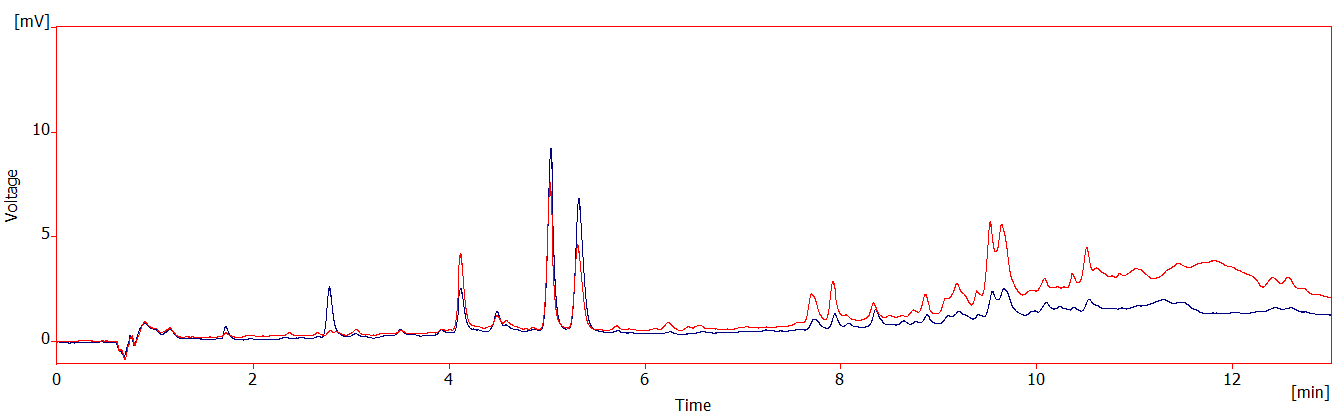


(b)

(ii)

(i)


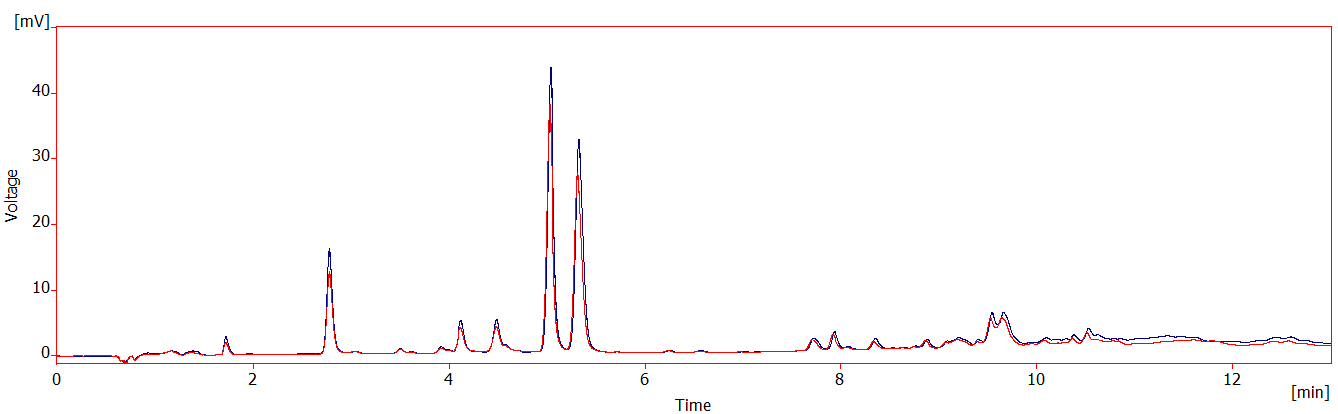


(c)


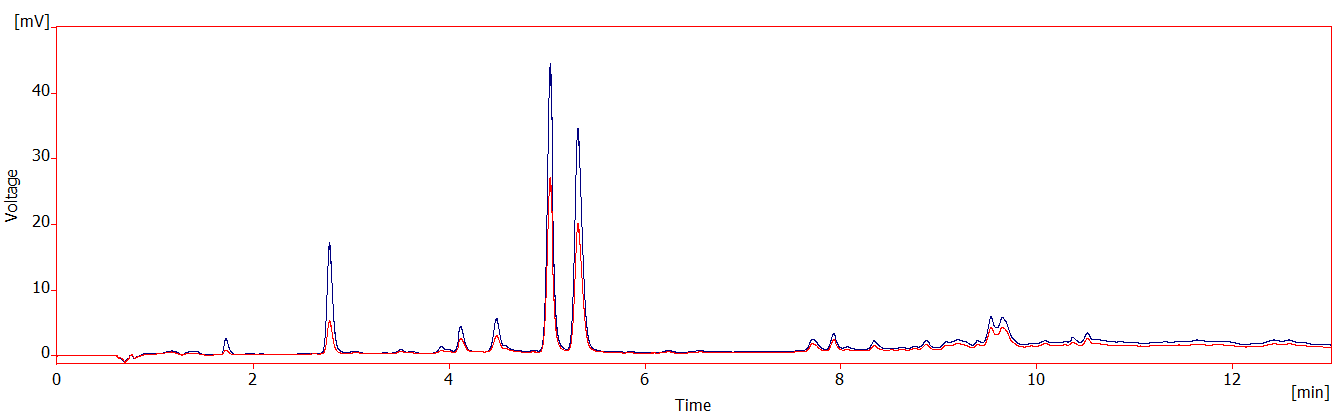


(e)

(d)

Supplementary Figure S2: Overlay of the HPLC (at 520 nm) chromatograms of the pellets of: **R2B + A/S medium:** (a) Controls (uninoculated) at t=0 (blue line) and t=24 h (red line) and (b) medium inoculated with *Pseudomonas* sp. R-27008 at t=0 (blue line) and t=24 h (red line); **MSM + A/S medium:** (c) Controls (uninoculated) at t=0 (blue line) and t=24 h (red line) and (d) medium inoculated with *Pseudomonas* sp. R-27008 at t=0 (blue line) and t=24 h (red line).

(e): PDA spectra of peak (i) (left) and peak (ii) (right) of the HPLC-DAD chromatogram (S1,b).
